# *Leishmania tropica *infection in wild and domestic canines

**DOI:** 10.1186/1756-3305-7-S1-O27

**Published:** 2014-04-01

**Authors:** G Baneth, A Nasereddin, Z Abdeen, CL Jaffe

**Affiliations:** 1 School of Veterinary Medicine, Hebrew University, P.O. Box 12, Rehovot 76100, Israel; 2 Al-Quds Nutrition and Health Research Institute, Al-Quds University, Abu Deis, Palestinian Authority; 3 Department of Microbiology and Molecular Genetics, IMRIC, Hebrew University-Hadassah Medical School, Jerusalem, Israel

## 

*Leishmania tropica *is a causative agent of cutaneous leishmaniasis in the Middle East, North Africa and some parts of southeastern Europe. It has also been described as a cause of human visceral leishmaniasis. Although cutaneous leishmaniasis caused by *L. tropica *is usually considered an anthroponotic infection transmitted between people directly by phlebotomine sand flies without the involvement of an animal reservoir, in Israel, Jordan and the Palestinian Authority it is a zoonosis with the rock hyrax (*Procavia capensis*) as a main reservoir host. Golden jackals (*Canis aureus*) and red foxes (*Vulpes vulpes*) have also been found to be infected with *L. tropica *in Israel and are assumed to have a role in spreading the infection to distant locations, but clinical signs of infection in these wild canids have not been detected.

In the domestic dog, *L. tropica *infection has been reported in only a few cases from Morocco and Iran where infection was mostly described as involving the visceral organs. While some surveys describe the detection of parasite infection from dog organs by culture or PCR without much detail on the manifestations of disease, reports from Morocco described two dogs infected with *L. tropica *with clinical manifestations similar to those found in canine *L. infantum *infection including generalized lymphadenomegaly, onychogryphosis, alopecia, keratoconjunctivitis, and also glomerulonephritis in one case. A report from northwestern Iran also described *L. tropica *in a dog with cutaneous and visceral involvement comparable to canine *L. infantum *infection. A young dog from a focus of *L. tropica *human cutaneous leishmaniasis near Jerusalem, with a large proliferative red mucocutaneous lesion between the upper lip and the nose was recently found to have mucocutaneous leishmaniasis caused by *L. tropica*.

In conclusion, domestic and wild canine infection with *L. tropica *may be more prevalent in areas of endemic human *L. tropica *cutaneous leishmaniasis than currently recognized, and canines should be evaluated as possible additional reservoirs for human infection.

## Funding

This study was supported by supported by the U.S. Agency for International.

Development (USAID) Middle East Regional Cooperation Program (MERC) project TA-MOU-08-M27-072 and by the Deutsche Forschungsgemeinschaft (DFG) as part of a German-Israeli-Palestinian cooperative project SCHO 448/8-1.

